# Health Care Professionals' Engagement With Digital Mental Health Interventions in the United Kingdom and China: Mixed Methods Study on Engagement Factors and Design Implications

**DOI:** 10.2196/67190

**Published:** 2025-04-04

**Authors:** Zheyuan Zhang, Sijin Sun, Laura Moradbakhti, Andrew Hall, Celine Mougenot, Juan Chen, Rafael A Calvo

**Affiliations:** 1 Dyson School of Design Engineering, Imperial College London, 25 Exhibition Road, London, SW7 2AZ, United Kingdom, 44 07568400428; 2 CW+, Chelsea and Westminster Hospital, London, United Kingdom; 3 West China Hospital, Sichuan University, Chengdu, China

**Keywords:** burnout, digital mental health interventions, engagement, eHealth, design, health care professional, health care workers, United Kingdom, UK, China, Chinese, occupational stress, mixed-methods, stigma, well-being, mental health, digital health, occupational health

## Abstract

**Background:**

Mental health issues like occupational stress and burnout, compounded with the after-effects of COVID-19, have affected health care professionals (HCPs) around the world. Digital mental health interventions (DMHIs) can be accessible and effective in supporting well-being among HCPs. However, low engagement rates of DMHIs are frequently reported, limiting the potential effectiveness. More evidence is needed to reveal the factors that impact HCPs’ decision to adopt and engage with DMHIs.

**Objective:**

This study aims to explore HCPs’ motivation to engage with DMHIs and identify key factors affecting their engagement. Amongst these, we include cultural factors impacting DMHI perception and engagement among HCPs.

**Methods:**

We used a mixed method approach, with a cross-sectional survey (n=438) and semistructured interviews (n=25) with HCPs from the United Kingdom and China. Participants were recruited from one major public hospital in each country.

**Results:**

Our results demonstrated a generally low engagement rate with DMHIs among HCPs from the 2 countries. Several key factors that affect DMHI engagement were identified, including belonging to underrepresented cultural and ethnic groups, limited mental health knowledge, low perceived need, lack of time, needs for relevance and personal-based support, and cultural elements like self-stigma. The results support recommendations for DMHIs for HCPs.

**Conclusions:**

Although DMHIs can be an ideal alternative mental health support for HCPs, engagement rates among HCPs in China and the United Kingdom are still low due to multiple factors and barriers. More research is needed to develop and evaluate tailored DMHIs with unique designs and content that HCPs can engage from various cultural backgrounds.

## Introduction

Health care professionals (HCPs) are one of the most vulnerable groups to occupational stress and burnout [1]. Burnout among medical staff has gradually increased over time and worsened during COVID-19 [2,3]. More studies have focused on digital mental health interventions (DMHIs) to alleviate HCPs’ stress and burnout, as they offer accessible and cost-effective solutions that circumvent many barriers commonly associated with traditional interventions [4-6]. Research in countries like the United States, Australia, and Europe has provided evidence that DMHIs based on mobile apps, websites, and virtual reality are accepted and can be efficacious among nurses, physicians, and medical trainees [4,7-12]. The majority of the literature has focused on apps and websites, with evidence on digital cognitive behavioral therapy, mindfulness interventions, mind-body and resilience skill training, and psychoeducational sessions delivered by mobile apps and websites [6,13]. Besides, virtual reality–based interventions have also begun to garner research focus, with recent preliminary studies demonstrating the effectiveness and high engagement among HCPs [14,15].

Despite the growing evidence of the effectiveness of DMHIs, lack of engagement was frequently reported in trials and studies [6,16]. Many recent reviews have shown that low engagement is a ubiquitous problem among DMHIs [17-19]. Plus, user engagement is considerably lower in naturalistic settings than in empirical studies [19-21]. To illustrate this, a review of 59 off-the-shelf mental health apps reported a median uptake rate of 4.0% and a 15-day retention rate of only 3.9% [22]. How to facilitate user engagement has become a priority in DMHI research [19], as a “sufficient dosage” is essential for DMHIs to achieve the intended outcomes [17,23,24].

Previous research has investigated the factors that impact user perception and engagement with DMHIs. Elements like help-seeking beliefs, therapist involvement, mental health, and digital literacy have been identified to affect the use of DMHIs [16,17]. Cultural-bounded factors like norms, beliefs, and stigma can also play a significant role in user engagement [16,21,25]. However, most studies on DMHI engagement were directed at patients and the general public [6,16,17]. Little research has focused on HCPs, who represent a unique population with distinct working contexts and are experiencing increasing levels of distress and burnout [22,26,27]. The level of engagement with DMHIs among HCPs and the factors that impact their engagement remain unclear.

This study aimed to address this research gap with a twofold objective: (1) to investigate HCPs’ engagement with and perceptions of DMHIs, (2) to identify key factors influencing HCPs’ engagement with DMHIs. In this study, we revealed several common factors among HCPs while exploring distinct cultural aspects that could affect DMHI engagement by investigating HCPs from 2 diverse countries: China and the United Kingdom. We also provide design and research implications for future studies on DMHIs for HCPs that could effectively engage end users and generate positive outcomes.

## Methods

We used a mixed method approach and collected qualitative (from semistructured interviews and open-ended questions in the survey) and quantitative data (from the survey).

### Recruitment

The study was carried out across 2 hospitals in China and the United Kingdom. The first hospital, West China Hospital, is in Chengdu, southwest China. The other is Chelsea and Westminster Hospital in London, UK. Both hospitals are publicly funded, and are among the largest ones in the local regions, providing comprehensive medical care to a high volume of patients, making them representative of large urban health care institutions in their respective countries. Although health care systems in China and the United Kingdom operate differently, some systematic challenges across the 2 hospitals are similar, such as staff shortages, increasing workload, and high burnout levels [27-30].

A volunteer sampling method was used for the survey. The link to the survey was spread to HCPs via email and group chats in WhatsApp and WeChat. To be eligible for the study, participants had to be over 18 years old and employed as staff members (both clinical and administrative) in one of the hospitals where the study was taking place.

Interview participants were recruited via the questionnaire. An option to enter their email address to be contacted for the interview; a separate interview information sheet and consent form were then emailed to participants.

### Study Design

#### Survey Design

The survey was designed to obtain demographic data and staff’s experience with and perception of DMHIs. The questions are tailored by the research team to the study objectives and focus on the following categories:

1. Demographic information, including gender, age, job role, and time being an HCP.
2. Mental health knowledge and help-seeking experience: single-item questions with text entries to swiftly assess if participants have knowledge of burnout and its interventions and if they have sought mental health support before.
3. Previous engagement with DMHIs: single-item questions to gauge participants’ experience with DMHIs, with text entry on DMHIs they have used before.
4. Willingness to engage with DMHIs: single-item question asking participants’ willingness to adopt DMHIs, with text entry asking their reason if unwilling to.

Before disseminating, the questions were reviewed by hospital administrators, mental health professionals, and digital health and HCI researchers to ensure the clarity, relevance, and appropriateness. A small group of 14 (6 in China and 8 in the United Kingdom) HCPs completed the survey as part of the pilot testing, and their feedback was used to refine the wording and structure of items to enhance comprehension and response accuracy.

#### Interview Design

The interview was planned to acquire deeper insights into the staff’s attitude and reason behind their amotivation to use DMHIs. Following a semistructured design, the interview questions covered the following topics:

1. Knowledge and perceptions of DMHIs.
2. Previous engagement with DMHIs and thoughts on the experience.
3. Motivation of uptaking DMHIs in the future and reasons behind it.
4. Suggestions and thoughts about their ideal DMHIs.
5. General thoughts on the topics covered (such as DMHIs, mental health, and technology).

#### Data Collection and Analysis

The web-based survey was collected and managed via Qualtrics and WJX (both are web-based survey platforms). Participants were explicitly informed about the survey content, data security measures, and their right to withdraw any time before entering the question page. All data were recorded anonymously. The data were securely extracted and analyzed using IBM SPSS Statistics for Mac software (version 26; IBM Corporation). Binary logistic regression was used to explore the contributing factors to HCPs’ past engagement with DMHIs, while *t* tests and *χ*² tests were used to investigate variances of groups. The level of significance was set at *P*<.05.

Interviews were carried out by the first author, who was trained in design and user study, via both face-to-face and web-based platforms (Zoom and Teams). Each interview lasted around 30 minutes. Participants were briefed about the study’s aim at the beginning of the interview. They were also informed of the data security measures of the study, and their right to withdraw from the study without any reason given. No relationship was established before the study commencement. No repeat interview was carried out. The authors identified and discussed data saturation, after which no further interview was scheduled.

Qualitative data were extracted from the audio transcription of the interviews and answers from open-text entries in the survey. Two bilingual researchers worked on extracting textual data. An inductive thematic analysis approach was used to collect the qualitative data from the interviews [31]. Two rounds of inductive thematic analysis were conducted by 2 bilingual researchers using NVivo for Mac (version 12; QSR International Pty Ltd). The qualitative data from each hospital were coded separately to preserve context-specific insights. Then, the research team compared and synthesized the codes to identify unique findings and shared themes across both hospitals.

### Ethical Considerations

The research has 2 parts in China and the United Kingdom. Studies in the 2 sites were approved by the Research Integrity and Ethics Committee of Imperial College London (22IC7803 and 22IC7585). The Ethics Committee on Biomedical Research of West China Hospital granted local ethics clearance for the research in China. Ethics approval was granted for the study in Chelsea and Westminster Hospital by the Health Research Authority of the National Health Service in the United Kingdom (reference number: 316935). Informed consent was obtained from participants before starting the survey and interviews. Participants were fully informed about the study's objectives, procedures, and potential risks. All data collected in the study was anonymized, and no personally identifiable information was linked to participant responses. No compensation was provided for completing the survey. However, participants completed the interview received a £5 voucher of appreciation for their time.

## Results

### Survey Findings

#### Demographics and Participant Characteristics

We gathered survey responses from 438 participants, 220 participants in China and 218 participants in the United Kingdom (Table 1). Given the diversity of the National Health Service workforce in the United Kingdom, we also inquired about ethnicity among the respondents. The ethnicity question was excluded as more than 98% of the workforce in the Chinese hospital is Han Chinese [32].

**Table 1.** Demographic data of the 2 samples.

	China (n=220)	United Kingdom (n=218)	Statistical comparison		*P* value
			*χ*² (*df*)	*t* test (*df*)	
Sex, n (%)			10.90 (3)	—^a^	.01
Female	186 (85)	159 (73)			
Male	34 (15)	55 (25)			
Others or prefer not to describe	0 (0)	4 (2)			
Age (years), mean (SD)	35.78 (8.26)	35.16 (11.25)	—	0.64 (410)	.53
Experience (years), mean (SD)	12.95 (8.88)	11.21 (10.27)	—	1.89 (437)	.06
Ethnicity, n (%)					
White	—	107 (49)	—	—	—
Asian	—	64 (29)	—	—	—
Black	—	25 (12)	—	—	—
Mixed and others	—	22 (10)	—	—	—
Job sector, n (%)			18.78 (2)	—	<.001
Doctor	107 (38)	64 (30)			
Nurse	84 (49)	103 (47)			
Others (admin, therapists, etc)	29 (13)	51 (23)			
Use of mobile phones at work, n (%)			27.05 (4)	—	<.001
Hardly ever	39 (18)	21 (10)			
Occasionally (once every 2‐3 hours)	38 (17)	31 (14)			
Sometimes (once every 1‐2 hours)	40 (18)	49 (23)			
Often (around once per hour)	53 (24)	53 (24)			
Very frequently (over 3 times per hour)	50 (23)	64 (29)			
Use of computers at work, n (%)			274.15 (4)	—	<.001
Hardly ever	15 (7)	8 (4)			
Occasionally (once every 2‐3 hours)	17 (8)	3 (1)			
Sometimes (once every 1‐2 hours)	14 (6)	15 (7)			
Often (around once per hour)	49 (22)	22 (10)			
Very frequently (over 3 times per hour)	125 (57)	170 (78)			
Know about burnout, n (%)	104 (47)	169 (78)	42.67 (1)	—	<.001
Know about burnout interventions, n (%)	66 (30)	142 (65)	55.65 (1)	—	<.001
Have sought mental health support before, n (%)	58 (26)	92 (42)	12.20 (1)	—	<.001
Have used DMHIs^b^ before, n (%)	14 (6)	62 (28)	37.21 (1)	—	<.001
Consider using DMHIs in the future, n (%)	184 (84)	155 (71)	9.83 (1)	—	.002

a Not applicable.

b DMHI: digital mental health intervention.

According to the *χ*² test results, significantly more HCPs in the United Kingdom had knowledge of burnout (*χ*²_1_=42.67, *P*<.001), its interventions (*χ*²_1_=55.65, *P*<.001), and had sought help on mental health before (*χ*²_1_=12.20, *P*<.001). Meanwhile, only 6% (14 out of 220) of Chinese participants reported having used DMHIs before, compared with 28% (62 out of 218) in the United Kingdom. This demonstrates a significantly higher past engagement with DMHIs among the UK cohort (*χ*²_1_=37.21, *P*<.001).

#### Factors That Predict HCPs’ Engagement With DMHIs

The results of the binary logistic regression are shown in Multimedia Appendix 1. In the UK sample, the regression model was statistically significant (*χ*²_11_=31.52, *P*<.001), indicating a good fit with the data (Nagelkerke R²=0.22, Hosmer and Lemeshow *χ*²_8_=4.61, *P*=.80). The results indicate that ethnicity was a significant predictor of DMHI engagement. Compared with HCPs identifying as white, those identifying as Asian (B=-1.69, *P*=.04, Exp(B)=0.18) and Black (B=-1.15, *P*=.01, Exp(B)=0.32) were significantly less likely to engage with DMHIs. Additionally, the odds of engaging with DMHIs were over twice as high for HCPs who had sought mental health support before (B=0.73, *P*=.045, Exp(B)=2.07) compared with those who had not. Furthermore, knowing about burnout interventions was a significant predictor of DMHI uptake (B=1.21, *P*=.02, Exp(B)=3.35). In the Chinese sample, the regression model was statistically significant (*χ*²_8_=15.57, *P*=.003), with Nagelkerke R²=0.18, Hosmer and Lemeshow *χ*²_8_=3.19 (*P*=.92). Having sought mental health support before significantly predicted HCPs’ prior DMHI use, with an odds ratio of 3.41 (B=0.89, *P*=.04).

To further explore the impact of mental health knowledge on DMHI engagement, we conducted *χ*² tests. In the Chinese sample, knowing about burnout interventions (*χ*²_1_ [n=220]=5.47, *P*=.02) and having sought mental health support (*χ*²_1_ [N=220]=7.30, *P*<.001) were significantly correlated with increased DMHI engagement. Although knowing about burnout is marginally significant (*χ*²_1_ [n=220]=3.50, *P*=.06). In the UK sample, knowing about burnout (*χ*²_1_ [n=218]=8.15, *P*=.004), knowing about burnout interventions (*χ*²_1_ [N=218]=13.39, *P*<.001), and having sought mental health support (*χ*²_1_ [N=218]=10.85, *P*<.001) were all significantly associated with higher DMHI engagement.

#### Attitudes Towards DMHIs

Only 6% (14 out of 220) of HCPs in China had engaged with DMHIs before, compared with 28% (62 out of 218) in the United Kingdom. Moreover, among the 6% of respondents in China, the “DMHIs” they mentioned using were often web-based mental health content published on WeChat, TikTok, and Bilibili (similar to YouTube).

Regarding their willingness to adopt DMHIs in the future, 16% (36 out of 220) of participants in China and 29% (63 out of 218) in the United Kingdom expressed a lack of willingness. It is notable that UK-based staff, although more experienced with DMHIs, were significantly less motivated to use DMHIs again (*t*=3.17, *P*<.001). Their reasons for unwillingness to use DMHIs were summarised and shown in Textbox 1, including low perceived need and usefulness, needing a break from screens, and negative prior experiences with DMHIs.

Textbox 1. **Reasons for their lack of motivation, provided by the health care professionals (HCPs) who were unwilling to use digital mental health interventions (DMHIs).**

**In China (n=36)**

- I don’t think it’s necessary (39%, n=14)
- I need a screen-break (19%, n=7)
- I can relieve my own stress (14%, n=5)
- I don’t know where to find suitable tools (14%, n=5)
- I worry about personal information leak (11%, n=4)
- Others (3%, n=1)

**In the United Kingdom (n=63)**

- I need a screen-break (22%, n=14)
- It not useful / doesn’t solve the real problem (21%, n=13)
- Phones distractive and can bring more stress (16%, n=10)
- It lacks a human touch (13%, n=8)
- I don’t think it’s necessary (9%, n=6)
- DMHIs I used were boring and not engaging (8%, n=5)
- Others (11%, n=7)

### Interview Findings

#### Overview

A total of 41 HCPs opted in for the interviews after finishing the survey. A total of 10 participants in China (comprising 3 doctors, 6 nurses, and 1 admin, with 7 females and 3 males) and 15 participants in the United Kingdom (4 doctors, 7 nurses, and 4 admins, with 10 females and 5 males, 7 identified themselves as White, 4 as Black, 3 as Asian, 1 as Mixed) finished the interview. A total of 16 staff members (6 in China and 10 in the United Kingdom) did not reply to follow-up messages or could not participate due to their intense schedules. Participants were anonymized and referred to in the form of “country - job type (D for doctors, N for nurses, and A for admins) – number” (eg, UK-D-01)*.*

The themes covered HCPs’ personal, contextual, and program-related factors. All quotes are included in Multimedia Appendix 2 and referred to by the quote numbers. Theme 1‐3 were shaped equally by qualitative data from both hospitals. Theme 4 reflected disparities between the 2 cohorts, with unique contributions from participants of each hospital.

#### Theme 1: No Problem, No Need

One common theme is a lack of perceived need to engage with DMHIs, with 2 primary causes as summarized below.

##### Subtheme 1.1: The Normalization of Burnout

Participants who were less aware of burnout mentioned getting accustomed to the extra pressure and stress in their daily work and having normalized such negative feelings as a common part of their job as HCPs (quotes 2 and 3 in Multimedia Appendix 2). As alluded to by a participant (quote 2 in Multimedia Appendix 2), such normalization of burnout is why many HCPs sought minimum support and believed support tools were unnecessary as they could handle stress and burnout by themselves.

##### Subtheme 1.2: Burnout Is Less Severe and Urgent

On the other hand, even those with a general understanding of burnout tended to underestimate its negative impact and thus deemed engaging with DMHIs unnecessary. This can be illustrated by participants who compared burnout with other psychological and physical conditions (quotes 3‐6 in Multimedia Appendix 2). For example, participants mentioned it is more necessary for colleagues or themselves who were undergoing difficult situations like a change of sex (quote 3 in Multimedia Appendix 2), PTSD (quote 4 in Multimedia Appendix 2), pregnancy (quote 5 in Multimedia Appendix 2), and depression (quote 6 in Multimedia Appendix 2) to use mental health apps. Burnout and other workplace issues were considered less problematic and less urgent (quotes 3 and 6 in Multimedia Appendix 2). Therefore, the perceived need for using DMHIs was relatively low.

### Theme 2: No Place for DMHIs

#### Subtheme 2.1: Lack of Time or Space to Use DMHIs

Due to their intense and unpredictable schedules and limited opportunities for breaks, participants mentioned it’s hard for them to use DMHIs routinely (quotes 7 and 8 in Multimedia Appendix 2), and they had to put much effort into finding a time and space to use DMHIs (quote 9 in Multimedia Appendix 2).

Furthermore, using DMHIs off work was less welcomed, and many preferred to leave work-related issues like stress and burnout at work (quotes 10 and 12 in Multimedia Appendix 2). Participants strongly valued work-life balance, preventing them from using DMHIs in their personal time (quotes 11 and 12 in Multimedia Appendix 2).

#### Subtheme 2.2: We Have a Lot of Screens Already

A big part of the participants (10 out of 25) mentioned the impact of hospital digitisation and its impact on their work (as represented by quotes 13‐15 in Multimedia Appendix 2). They recalled the extra stress and workload (quotes 13 and 14 in Multimedia Appendix 2) and a sense of losing control over their work (quotes 13 in Multimedia Appendix 2) due to the introduction of new digital tools in the health care system. The negative experience with digital systems could lead to extra hesitation when HCPs adopt DMHIs. Besides, the ubiquity of digital systems in the hospital led to HCPs’ need for a screen break, which participants highlighted as a hindrance to their motivation to use DMHIs (as represented by quote 15 in Multimedia Appendix 2).

### Theme 3: Mixed Perceptions of DMHIs

#### Subtheme 3.1: DMHIs Are Good for Relaxation and Psychoeducation

When asked about their expectations of DMHIs, most participants (17 out of 25) desired the destress and relaxation sessions offered by DMHIs. Participants generally praised the potential of DMHIs for quick relaxation and stress relief (as represented by quotes 16 and 17 in Multimedia Appendix 2). Several participants also mentioned their preference for psychoeducational content, such as mental health tips, assessment, skill training, and general wellbeing suggestions (quotes 18 and 19 in Multimedia Appendix 2).

#### Subtheme 3.2: Need for Relevant and Reliable Content

Participants in China mentioned their generally negative experiences with mental health tools and web-based platforms (as represented by quotes 20 and 21 in Multimedia Appendix 2). They recalled getting mental health information via social media platforms, like WeChat, Douyin (TikTok) and Zhihu (similar to Quora). Due to the complex and unverified sources of information, such content was often not trustworthy (quote 20 in Multimedia Appendix 2) and superficial (quote 21 in Multimedia Appendix 2).

Meanwhile, participants in both sites highlighted that many DMHIs they encountered failed to address the specific and complex needs of HCPs (as represented by quotes 21‐24 in Multimedia Appendix 2). Participants were dissatisfied that many DMHIs only offered broad and generic support (quotes 23 and 24 in Multimedia Appendix 2). They emphasized the need for more personalized and relevant solutions tailored for HCPs (quotes 22 and 24 in Multimedia Appendix 2).

#### Subtheme 3.3: Desire for a Human Touch

A majority of interviewees (11 out of 15) in the United Kingdom highlighted their preference for a human touch in mental health support. Lack of human support emerged as a critical hindrance in their motivation to use DMHIs. For instance, one participant mentioned their preference for interacting with a real human being and being able to vocalize their thoughts rather than only focusing on their phones (quote 25 in Multimedia Appendix 2). Other participants mentioned similar points and further elaborated on the significance of persons who genuinely understand the daily situations of HCPs and can immediately relate to their problems (quotes 26 and 27 in Multimedia Appendix 2).

The need for a personal touch was less brought up by Chinese participants. Two participants briefly mentioned the need for expert support to “suggest on whether I have a mental situation” [CN-N-04], and “give personalized guidance and stress relief suggestions” [CN-N-02].

### Theme 4: Culture and Stigma

#### Subtheme 4.1: Self-Stigma and the Moral Obligation Among Chinese HCPs

The impact of self-stigma on DMHI engagement was apparent among Chinese participants. For example, one participant thought DMHIs were unnecessary because HCPs should be able to address their own problems (quote 28 in Multimedia Appendix 2). Other participants displayed similar attitudes and associated personal and professional virtues with mental well-being (quotes 30 and 31 in Multimedia Appendix 2). Such moral attribution was shared by other participants who used terms like “not strong enough*”* (CN-N-06) and *“*not trying hard enough*”* (CN-N-01) to describe HCPs struggling with stress, anxiety, and burnout.

Furthermore, as described by participants (quotes 29‐31 in Multimedia Appendix 2), participants in the Chinese hospital often share a moral obligation to improve themselves and stay strong and resilient despite experiencing high levels of stress and burnout. As reflected by the quotes, such moral commitments can contribute to their resilience while leading to their focus on self-improvement rather than seeking external support.

#### Subtheme 4.2: Individualism and Collectivism, Reversed

As conceptualized by Hofstede [33], in individualistic countries like the United Kingdom, people tend to prioritize self-care over seeking help from in-group members [33,34]. In contrast, in a collectivist culture, collective growth and harmony are prioritized [35]. However, in this study, we identified the opposite trends regarding preferences for DMHIs among participants. Specifically, more participants in the UK-based-hospital preferred community support and human involvement in DMHIs (as represented by quotes 32 and 33 in Multimedia Appendix 2). They were generally open to seeking support from their local community (quote 32 in Multimedia Appendix 2), and work community (quote 33 in Multimedia Appendix 2), and deemed providing such support opportunities a valuable feature of DMHIs.

In the Chinese hospital, however, few participants expressed similar interests in community support. One participant described a reluctance to community support and team building among younger generations of HCPs (quote 34 in Multimedia Appendix 2). Other participants strongly preferred personal support, self-care, and self-improvement (as represented by quotes 29 and 35 in Multimedia Appendix 2). Regarding social support, they preferred to seek help within their personal support network instead of from broader groups and communities (quotes 35‐37 in Multimedia Appendix 2). As revealed by quotes 36 and 37 in Multimedia Appendix 2, when asked about community support options facilitated by DMHIs, Chinese participants often envisioned situations where they needed to disclose sensitive and private information, which was less favored.

## Discussion

### Principal Findings

To our knowledge, this is one of the first studies investigating HCPs’ use and factors impacting their engagement with DMHIs. Based on their feedback, we identified demographical, personal, occupational, and cultural factors that impacted their engagement with DMHIs and provided implications for future research on DMHIs for HCPs. In the following sections, we elaborate on the engagement factors, compare our findings with prior works on DMHI engagement among other populations, and provide design implications.

Furthermore, building on the findings of this study and evidence from previous literature, we developed detailed design heuristics and practical recommendations for DMHIs targeted at HCPs, as outlined in Multimedia Appendix 3.

### Demographic Factors: Lack of Engagement Among Underrepresented Groups

In this study, we identified substantial differences in HCPs’ engagement with DMHIs across cultures and ethnicities. Specifically, only 6% of HCPs in China had previously used DMHIs, compared with 28% of staff in the United Kingdom. Furthermore, within the UK sample, HCPs belonging to ethnic minority groups, specifically Asian and Black, were significantly less likely to engage with DMHIs. This concerning gap in DMHI engagement among ethnic minorities and people from low- and middle-income countries aligns with findings from previous studies [36,37]. One fundamental cause of such lack of engagement is that most mental health apps and platforms are developed in high-income countries for the majority of the White population [25,38,39]. This poses barriers and challenges for users from underrepresented groups to engage. In our study, HCPs in China struggled to find available DMHIs and were frustrated by the lack of quality of the existing products and platforms (subtheme 3.2). Such inequality in resources, along with language barriers and cultural incompatibility, were major factors identified in previous studies that hinder engagement from underrepresented populations [25,40,41]. More efforts are strongly needed to develop tailored interventions [42] and conduct cultural adaptation on existing DMHIs for underrepresented populations [25,43,44] to overcome this inequality and facilitate engagement among underrepresented groups.

### Personal Factors: Mental Health Literacy and Low Perceived Need

Lack of mental health knowledge and low perceived need emerged as interconnected factors hindering DMHI engagement in both samples. Our results showed that knowledge of burnout interventions and a help-seeking history significantly predicted HCPs’ engagement with DMHIs. Many HCPs, without adequate knowledge and recognition of burnout, had normalized burnout symptoms in their work experiences (subtheme 1.1) and found it less necessary to adopt DMHIs for burnout as it is less severe and urgent (subtheme 1.2). Indeed, without essential literacy and awareness, motivating users to adopt DMHIs can be challenging [45]. This is especially true among HCPs in various stages of burnout. Previous studies have shown that the need to alleviate acute symptoms is a crucial motivation for users to engage with DMHIs [16,21,46]. For HCPs experiencing burnout, however, such motivation is often lacking, as burnout tends to gradually deteriorate over time rather than present acute issues requiring immediate relief [1,3]. Hence, to facilitate early recognition of burnout and motivate HCPs to seek support, systematic psychoeducation for staff is recommended for health care institutions, which was also called for by other studies [47,48]. Moreover, consistent with the design heuristics proposed by Peters [49], we recommend future studies on DMHIs for HCPs to provide adequate information on the necessity and benefits of interventions across various stages of user experiences. This can help form a meaningful rationale among HCPs who are hesitant about engaging or just started using DMHIs, which can effectively motivate them to increase and sustain their engagement [49,50].

### Occupational Factors: Demanding Jobs and Overwhelming Digitization

As revealed by previous literature, engagement is critical for effective DMHIs [19,51,52]. However, this can be challenging for HCPs as their schedules are intense and unpredictable. HCPs in China and the United Kingdom were reported to undergo issues like staff shortages, increasing workload, and work pressures [29,30]. This is also reflected in this study, HCPs struggled to allocate a set time to engage with DMHIs (subtheme 2.1). For them, structured push notifications, a common engagement strategy applied by prior studies [53-57], may be less effective. However, just-in-time notifications, that is, adaptive and personalized notifications based on user behavior patterns or their locations [58]**,** demonstrated better engagement outcomes [58,59] and can be an ideal engagement facilitator for HCPs. Although HCPs’ schedules vary significantly, certain times (morning brief, between shifts, lunch breaks, etc) can be suitable for specific cohorts. Furthermore, as HCPs’ work is highly location-based, we suggest using context-based reminders to facilitate engagement. For instance, just-in-time notifications that prompt when HCPs enter spaces used for relaxation and on-site visual cues like posters and stickers placed within common spaces could be effective nudges for DMHI engagement. Similar to the design strategies proposed by previous studies that support establishing an engagement routine [60], such context-based reminders could promote the integration of HCPs’ existing routines with DMHI engagement.

Digitization in health care systems is another factor that cannot be ignored. Our quantitative data revealed that a considerable proportion of participants (88% in China and 79% in the United Kingdom) reported using computers frequently (at least once an hour) in their jobs. Being immersed in a highly digitized workplace, participants in both countries reflected on the additional effort and learning curve entailed by systems like the electronic health record. They demonstrated hesitance and reluctance to engage with more digital systems like DMHIs, with a strong need for a “screen break” (as shown in the survey feedback and Subtheme 2.2). This is consistent with recent studies on digital stress and overload among HCPs [61], often caused by digital systems like electronic health records that have been ubiquitously applied in health care settings [62-65]. Concerns of being further overwhelmed and complicated by technology contribute to their hesitation and lack of motivation towards DMHIs. Therefore, we suggest that future work apply best practices for usability and accessibility to avoid causing an extra burden on HCPs’ work experience, a point also advocated by other researchers [7,49,66]. We also recommend that researchers and designers consider HCPs’ digital literacy (especially for older, non-tech-savvy staff, as suggested by prior studies [64,67] and deliver DMHIs via technologies familiar to the targeted cohort of HCPs.

### Intervention-Based Factors: Relevance and Human Support

Many participants indicated that DMHIs often lacked relevance to health care workers and their struggle with job burnout. Similar observations have been made in other studies, where users strongly valued personal relevance, highlighting the importance of tailoring to DMHIs [16,17]. To provide relevant content and design for HCPs, we recommend incorporating language and daily scenarios that staff are familiar with. Moreover, using participatory design methodologies [68] to involve HCPs and relevant stakeholders throughout the developing process is also recommended to ensure the final outcome embodies the values and needs of HCPs, a perspective shared by many DMHI researchers [69-71].

Besides, staff also mentioned a need for human-based support, highlighting the importance of feeling understood and related to their experiences with DMHIs (subtheme 3.3). Prior studies also suggested the benefits of providing human-based support or guidance, like expert consultation and coaching, to promote user engagement [36,52,56,72,73]. DMHIs with certain levels of human support generally result in better engagement and clinical outcomes than self-guided DMHIs, as reported by recent reviews [19,73]. However, expert guidance and regular meetings with trained practitioners are less accessible and scalable, especially for large populations like HCPs [19]. We suggest future studies probe the potential of accessible human support and guidance, like text-based, asynchronized human support, or human-like interactions supported by conversational AI. Indeed, DMHIs using chatbots and embodied conversational agents have the potential to facilitate user engagement by providing personalized and empathic interactions [74,75]. Nonetheless, more studies are needed to address the ethical and methodological shortfalls [76,77] and tailor the algorithms for unique cohorts like HCPs.

### Cultural Factors: Stigma, Moral Obligation, and Varying Preferences for Community Support

Cultural differences are influential to HCPs’ engagement with DMHIs, as revealed by our qualitative data. Stigma on burnout and mental health issues was commonly identified among Chinese participants, echoing research evidence on the prevalence of stigma in China [78,79]. The self-stigma among Chinese participants affected their willingness to admit mental health issues or adopt DMHIs for support (subtheme 4.1). Previous studies among culturally diverse populations also emphasized the impact of self-stigma on DMHI acceptability and engagement [21,41]. Furthermore, Chinese HCPs in this study often attributed mental health conditions to personal and moral virtues, demonstrating a moral obligation to remain resilient at work and to do their best to avoid being affected by stress and burnout (subtheme 4.1). Such moral experiences are closely intertwined with culturally rooted mental health stigma and are commonly observed within Chinese communities and other cultural groups [80,81]. Maintaining the ability to work is a crucial coping strategy among Chinese populations against mental health stigma and sociocultural discrimination, as work serves as a key factor in preserving social status and fulfilling familial and moral obligations [28,81]. We suggest future projects for stigmatized populations establish nonjudgmental feedback mechanisms and reframe DMHIs as tools for enhancing well-being, occupational resilience, or improving professional abilities and skills, a strategy also indicated by a recent study on DMHIs [67]. Although researchers suggested shifting focus away from the dichotomous categorization of wellness and health within digital mental health technologies [82], we argue that strategically labeling DMHIs can be beneficial in a stigmatized context.

Peer support and community-based elements in DMHIs could facilitate user engagement by fostering a sense of collective care and support [83]. However, as suggested by our findings, this must be executed cautiously, particularly in environments where mental health is still stigmatized and less discussed openly. Similar to Chinese HCPs in this study, stigmatized users are often reluctant to participate in community support activities [84,85], due to sociocultural factors like public stigma, lack of mental health education, and traditional cultural values [86-88]. We suggest designers build meaningful connections among users while not imposing pressure to share or disclose personal information, which is crucial for nurturing social connectedness and support [49,50]. Maintaining a certain level of anonymity and taking extra caution with user privacy is also advisable.

### Limitation

This study involved 2 major hospitals in key cities in China and the United Kingdom. Given the differences between hospitals of various sizes and geographical locations [89], our findings may not fully represent other medical populations in these countries. Our sample sizes for the survey and interview (around 5% of survey respondents) are relatively small, which may limit the generalizability of our findings. Besides, the gender imbalance between the 2 cohorts (*χ*²_3_=10.907, *P*=.01) may have also impacted our findings, as gender can be an impactful factor on DMHI engagement [16].

The volunteer sampling method we employed could also introduce a source of response bias. HCPs interested in mental health and DMHIs may have been more likely to take part. This may lead to the study results not being fully generalizable to the broader population of HCPs in China and the United Kingdom. However, the triangulation of surveys and interviews helps somewhat mitigate this potential bias. Furthermore, as a cross-cultural study, we only investigated HCPs in 2 countries. Future studies should broaden the scope to include participants from other countries and regions to enhance the robustness of the evidence.

Participants were asked about DMHIs as a general, nonspecific concept rather than specific digital tools (eg, apps, web-based platforms, mixed-reality). This was intentional, as our focus was on overall engagement with various DMHIs. However, this broad framing may be less effective in helping HCPs navigate different DMHI concepts. More studies on engagement with specific types of technologies could be beneficial.

Another limitation of this study is the use of single-item questions assessing some constructs like engagement with DMHIs and willingness to adopt them. Although such questions were adopted to ensure the time-efficiency of the survey as HCPs are known to have hectic work schedules, they may lack psychometric stability on measures like motivation and willingness. Future research should consider using multi-item scales to enhance the robustness of measurement.

### Conclusions

This study used a mixed method approach to explore HCPs’ level of engagement with DMHIs and identified key factors that impact their attitudes and engagement. We found that there is a considerable gap in DMHI engagement among ethnic and cultural minority groups, potentially due to the lack of availability and content quality of DMHIs for minority groups. Lack of time to use DMHIs, lack of mental health literacy, low perceived need, lack of perceived relevance, and lack of human-based support were common factors impeding HCPs’ engagement with DMHIs. Cultural elements like stigma and moral attributions also impacted HCPs’ engagement and preference for DMHIs. Overall, this study contributed to the limited evidence on the experience and engagement with DMHIs among HCPs and shed light on how future efforts can design and deliver DMHIs that are accepted and engaged by HCPs.

## Supplementary material

10.2196/67190 Multimedia Appendix 1 Logistic regression analysis of digital mental health intervention engagement among health care professionals in China and the United Kingdom.

10.2196/67190 Multimedia Appendix 2 Themes, subthemes, and selected quotes.

10.2196/67190 Multimedia Appendix 3 Key design recommendations according to engagement factors.

## Abbreviations

DMHI: digital mental health intervention
HCP: health care professional
