# Supplementary material for: Health Care Professionals' Engagement With Digital Mental Health Interventions in the United Kingdom and China: Mixed Methods Study on Engagement Factors and Design Implications
Source: JMIR Ment Health. 2025 Apr 4;12:e67190. doi: 10.2196/67190 (PMC11990651; doi:10.2196/67190)
Supplement: Multimedia Appendix 1 [file mental-v12-e67190-s001.docx]

Table S1. Logistic regression analysis of DMHI engagement among HCPs in China and the UK

| Groups | Variables | B | S.E. | Wald | Sig. | Exp(B) | 95% CI Lower | 95% CI Upper |
| --- | --- | --- | --- | --- | --- | --- | --- | --- |
| **UK** | Gender | -.015 | .320 | .002 | .963 | .985 | .527 | 1.843 |
|  | Age | .009 | .027 | .123 | .726 | 1.009 | .958 | 1.063 |
|  | Ethnicity |  |  | 9.566 | .023 |  |  |  |
|  | Ethnicity (Asian) | -1.693 | .829 | 4.170 | .041 | .184 | .036 | .934 |
|  | Ethnicity (Black) | -1.149 | .461 | 6.218 | .013 | .317 | .128 | .782 |
|  | Ethnicity (Mixed & Others) | -.662 | .611 | 1.176 | .278 | .516 | .156 | 1.707 |
|  | Job Sector |  |  | 1.137 | .566 |  |  |  |
|  | Job Sector (Nurse) | .024 | .416 | .003 | .953 | 1.025 | .454 | 2.314 |
|  | Job Sector (Others) | -.475 | .523 | .827 | .363 | .622 | .223 | 1.731 |
|  | Experience | -.027 | .031 | .757 | .384 | .974 | .917 | 1.034 |
|  | Have sought mental health support before | .728 | .362 | 4.037 | .045 | 2.071 | 1.018 | 4.214 |
|  | Know about burnout | .326 | .594 | .301 | .583 | 1.385 | .433 | 4.433 |
|  | Know about burnout Intervention | 1.208 | .503 | 5.766 | .016 | 3.348 | 1.249 | 8.977 |
| **China** | Gender | -.752 | 1.073 | .491 | .484 | .471 | .058 | 3.864 |
|  | Age | -.184 | .113 | 2.672 | .102 | .832 | .667 | 1.037 |
|  | Job Sector |  |  | .104 | .949 |  |  |  |
|  | Job Sector (Nurse) | .240 | .761 | .100 | .752 | 1.271 | .286 | 5.649 |
|  | Job Sector (Others) | .140 | 1.063 | .017 | .895 | 1.150 | .143 | 9.245 |
|  | Experience | .101 | .093 | 1.186 | .276 | 1.107 | .922 | 1.329 |
|  | Have sought mental health support before | 1.227 | .596 | 4.246 | .039 | 3.411 | 1.062 | 10.960 |
|  | Know about burnout | .893 | .773 | 1.337 | .248 | 2.444 | .537 | 11.113 |
|  | Know about burnout interventions | .693 | .679 | 1.042 | .307 | 1.999 | .529 | 7.561 |
