# Supplementary material for: Health Care Professionals' Engagement With Digital Mental Health Interventions in the United Kingdom and China: Mixed Methods Study on Engagement Factors and Design Implications
Source: JMIR Ment Health. 2025 Apr 4;12:e67190. doi: 10.2196/67190 (PMC11990651; doi:10.2196/67190)
Supplement: Multimedia Appendix 2 [file mental-v12-e67190-s002.docx]

Table S2. Themes, subthemes and selected quotes

| **Themes** | **Subthemes** | **Select Quotes** |
| --- | --- | --- |
| Theme 1: No problem, no need | Subtheme 1.1:The normalisation of burnout | Quote 1: *“You know, all the stress, fatigue and mental pressure are quite common in the medical field … but if you can accept your job proactively, it might solve the problem … I’m not sure how many people will think they need to use an app for such stress.”* (CN-D-02).  *Quote 2: “… And then my daughter finally said to me, Mom, even when you're home, you're not home. I think that was the wakeup call, the moment of epiphany for me ... I think that's the problem with burnout is that you* *always become accustomed to that extra pressure, that extra expectation that there is on you. That by the time you realise that you can't do this anymore, it's too late ... And the level of support you seek is minimal. Because you’ve normalised it and everyone, including yourself thought that you'd been handling it.” (UK-N-05)* |
|  | Subtheme 1.2: Burnout is less severe and urgent | Quote 3: “So we have the staff mental health app. I've seen it a few times on our communication board. I haven’t tried it myself, but I know that some colleagues have used the app for mental health related situations. Like, I know a colleague who is going through a change in sex and has approached the app and used it for support. But I haven’t used it myself, as I’m not really in these difficult situations.” (UK-A-02)  Quote 4: “For some situations, especially like PTSD and another sort of mental disorder, staff can go to speciality hospitals and try to solve them. But if it’s for stress and burnout at work, I think it’s common for everyone to find their own way to cope with it.” (CN-D-01).  Quote 5: “The only time I ever tried anything was when I was pregnant, I used an app then for sleep. And that was good, since I was having difficulty sleeping. But besides that, I have to be honest with you, I haven't tried any digital apps for stress or burnout, no.” (UK-D-04).  Quote 6: “I know there are websites for depressive individuals to self-assess and that, but for burnout and stress I think, it seems not that urgent … I’m not sure how many people will go online for help.” (CN-N-03). |
| Theme 2: No place for DMHIs | Subtheme 2.1: Lack of time and hard to engage routinely | Quote 7: “There’s no one who can say like, I have a regular routine of breaks. Our schedule is so dependent on the patients of each day” (CN-D-03)  Quote8: “Sometimes our break is so short, and using the toilets, drinking some water, and stretching yourself will be all you can do... So it’s hard (to use any DMHIs regularly).” (CN-N-02) |
|  |  | Quote 9: “I still use an app now. I just always have to try and give myself some time to find a space … it’s hard, especially if you're at work with patients and families around” (UK-N-03).  *Quote 10: “I would prefer not to open any apps or content about work as possible at home. I am … (name) the nurse at work, and I’ll be … (name) the Mom at home.”* (UK-N-05)  *Quote 11: “… neither me nor most of my colleagues who have young children could add additional things to their off-work time. If we do anything, it has to be within our working hours.” (UK-D-02)*  *Quote 12: “When I am at work, I think of work-related stuff all the time. But when I am off work, I would like to have my own personal space, I would rather enjoy just using Douyin (Tik-Tok) and watching TV dramas than using a work-related app.” (CN-N-06)* |
|  | Subtheme 2.2: We have a lot of screens already | *Quote 13: “But sometimes Cerner^^[[1]](#footnote-1)^^ is making things more difficult. With the whole digitalisation, us trying to find ways around Cerner, trying to navigate that … is, I would say complicated, and not very reliable.” (UK-D-02)*  *Quote 14: “For us, we went live with Cerner in, around November 2019, and then March 2020 was the pandemic, which is not a long period of time for embedding. And that was particularly stressful for some of us.” (UK-N-07).*  *Quote 15: “Instead of using an App to relax, I would probably just close my eyes, or walk around to relax, as I often stare at the screens too much during each shift and my eyes sores.” (CN-D-01).* |
| Theme 3: Mixed perceptions of DMHIs | Subtheme 3.1: DMHIs are good for relaxation and psychoeducation | *Quote 16: “... almost everyone working in the hospital experiences high stress, most of the time we just need a quick relief of the stress.”* (CN-N-06).  Quote 17: “Quick relaxation and music sound good. I have an app that I use every day with the mindfulness sessions on. So that's really good as well.” (UK-N-03).  *Quote 18: “… these sorts of mental health scales are quite good as it allows you to know your current mental status”* (CN-D-01).  Quote 19: *“Mental health tips are good, or an assessment, things like that would be very helpful. Sometimes it's that skill, when you find it hard to express yourself in the room the correct way. And the other person can understand if equipped with listening skills. I think we all think we're great listeners but we could all need a bit of help with that.” (UK-A-01)* |
|  | Subtheme 3.2: Need for reliable, relevant and specific content | *Quote 20: “If an online tool can provide, for instance, a systematic evaluation like those in a hospital, that can indeed be useful. But from my experience, firstly there's no tool available for things like burnout ... and usually it’s full of ads and click baits...” (CN-N-05)*  *Quote 21: “I tried to open some of the websites that do personality and mental health tests as they seemed quite interesting, but then they don’t offer too much apart from general guidance.” (CN-A-01)*  *Quote 22: “My concern with these online tools probably … they’re not specific enough. I think the basic things we all know, you can just go on the NHS website and you can find mental health advice that it's already there … I think what is needed really is something more personalised.” (UK-D-02)*  *Quote 23: “For online self-help … I suppose my perception would be that you have to be quite self-motivated to do it. And a lot of these online things, I think they're quite generic in terms of the answers that they give...” (UK-D-01)*  *Quote 24: “I think there are enough apps and websites and mental health tips and advice. The problem is not accessibility anymore, I think often as healthcare staff our problems are more complex. Current digital tools, and mental health tips are not enough … like talk to your manager, or keep writing a diary, either people know them already but don't do them, or it feels to them that it's not really addressing their issues.” (UK-A-02)* |
|  | Subtheme 3.3: Desire for a human touch | *Quote 25: “I just like sharing thoughts verbally with somebody out there, even if it’s virtual. Yeah, so none of those things even needed to be face to face, even if it was on Zoom, like as we are talking now. I still find just better comfort in vocalising and speaking with somebody rather than an app.” (UK-N-05)*  *Quote 26: “But if there's a very individual component like, you're particularly stressed about certain type of thing you do in the workplace, or an issue with a colleague … when it comes to the nuances, that’s when things like apps don’t help so much because you just don't have another person who can directly respond to the things that you are worried of.” (UK-D-01)*  *Quote 27: “… sometimes we tend, if it is work-related, to go to our psychologists, because as I said,* *neonatology is such a tiny, specialised sector that is not like any other ward, it is so specific. That’s like talking to a person who immediately understands the situation.” (UK-A-02)* |
| Theme 4: Culture and stigma | Subtheme 4.1: “We don’t need help” - self-stigma and the moral attribution | *Quote 28: “Like my colleagues, they should be able to adjust* *themselves and relieve* *their own stress ... you need to have this ability of self-control as a healthcare professional ...” (CN-D-01).*  *Quote 29: “I used to feel stressed out and became anxious during work. But sometimes you just have to stick to your job and, force yourself to be stronger, and improve yourself in your professional skills and other abilities. It’s this sort of constant self-improving that helps me deal with such stress and anxiety.” (CN-N-05).*  *Quote 30: “Even if I feel burnt out, I just have to do what I should do. I think this is a matter of professional ethics. No matter what's happening or whatever your emotional state is, you should conduct yourself in this professional manner at work.” (CN-D-03).*  *Quote 31: “**I don’t know about doctors, but for us nurses I think 9 out of 10* *have burnout ... as I work as a healthcare professional, it’s part of my job, and if I let that (burnout) affect my mental health and my life, that would be unnecessary.” (CN-N-03)* |
|  | Subtheme 4.2: Individualism and collectivism, reversed | *Quote 32: “I always think community supports are really great … If, like in an app, you find that demographic information, like, how many people within the trust live in your area ... It allows people to then use that kind of community aspect of your area.” (UK-A-03).*  *Quote 33: “I think in the work environment, the sense of community is really what I’m looking for in terms of my health and wellbeing. Feeling like I’m part of the community, then I have a motivation to work better to get on better with people to work as part of a team. I think when it comes to wellbeing, anything involved with creating a community atmosphere is something I would benefit from.” (UK-A-04)*  *Quote 34: "Community support, like team building, which used to be very popular in our generation ... But now, with people born in the 90s and 00s entering the workforce, they are quite opposed to this and don't want any team activities during their personal time. ... they prefer to be alone, not hanging out with colleagues. " (CN-D-01)*  *Quote 35: “I usually take some time off, spend time by my own, zone out and things get better. I wouldn’t say that I enjoy disclose too much to my colleagues about my mental health situations” (CN-N-03).*  *Quote 36: “Privacy and confidentiality is a major concern ... I would rather chat with friends in private, but not to disclose much sensitive information online or to my work community.” (CN-D-03)*  *Quote 37: “When I’m off work, I just vent to my mates about everything at work … If you have mental health illnesses, you simply don’t want anyone at your work to know about this stuff.” (CN-A-01)* |

1. A EHR system applied broadly in the NHS (https://digital.nhs.uk/services/digital-and-interoperable-medicines/resources-for-health-and-care-services/list-of-epma-suppliers/cerner) [↑](#footnote-ref-1)
