# Supplementary material for: Health Care Professionals' Engagement With Digital Mental Health Interventions in the United Kingdom and China: Mixed Methods Study on Engagement Factors and Design Implications
Source: JMIR Ment Health. 2025 Apr 4;12:e67190. doi: 10.2196/67190 (PMC11990651; doi:10.2196/67190)
Supplement: Multimedia Appendix 3 [file mental-v12-e67190-s003.docx]

Table S3. Key design recommendations according to engagement factors

| Engagement Factors | Design Heuristics | Practical Design Recommendations |
| --- | --- | --- |
| Personal factors | - Provide psychoeducational content for HCPs | - Inform HCPs facts about burnout even before their engagement with DMHIs, using educational materials, campaigns, and in-person webinars or workshops.  - During their engagement with DMHIs, offer easily digestible, modular educational content on burnout, as preparation activities or wrapping-up tips.  - Incorporate stories or testimonials from fellow HCPs who have experienced burnout. Peer-shared narratives can help increase awareness, reduce stigma without putting pressure on stigmatised HCPs. |
|  | - Provide meaningful rationale in every step of user experience | - Provide information on the necessity of activities, especially those that need increased user efforts.  - Clearly demonstrate the benefits, gains and growth after each activity or session, to provide positive engagement and encourage future use. |
| Intervention-based factors | - Provide tailored programmes for HCPs’ focusing on issues like burnout | - Incorporate language and scenarios that are familiar to HCPs.  - Use characters that represent specific cohorts of HCPs to make them feel relevant and represented.  - Highlight the values commonly shared by HCPs, such as providing quality patient care, work-life balance, resilience and responsibility. |
|  | - Involve HCPs and stakeholders like resident psychologists in the process of designing and developing DMHIs | - Use participatory design or co-design methods and toolkits to stimulate idea generation and exchange among diverse groups of stakeholders. |
|  | - Provide human support | - If feasible, incorporate human-based support, provided by resident psychiatrists or occupational wellbeing specialists.  - If human support is less accessible within the hospital, provide asynchronous support provided by trained practitioners from a certified third-party organisation.  - Incorporate conversational AI for daily engagement guidance, structured Q&A, and basic conversations, while be mindful on the applicability and biases in the training dataset. |
| Occupational factors | - Explore just-in-time (JIT) notifications for HCPs to encourage engagement. | - Designers should understand HCPs’ daily routines and work with different cohorts of HCPs to design JIT notifications.  - Allow personalisation of the JIT notification patterns.  - Create context- or site-specific JIT reminders in spaces where it’s suitable for HCPs to relax and use DMHIs. |
|  | - Develop strategies that integrate DMHIs into HCPs’ routines | - Seek for common patterns of how HCPs take a break and try to encourage DMHIs engagement based on it (e.g. facilitate regular engagement during their lunch breaks) |
| Demographical and cultural factors | - Position the intervention as self-care or wellbeing support for cultures with stronger self-stigma | - Highlight the value of DMHIs in improving oneself in terms of professionalism and resilience.  - Be mindful of judgemental and evaluative languages and elements  - Apply additional care on user data privacy and make explicit claims on data safety |
|  | - Tailor DMHIs or use cultural adaptation methods for underrepresented populations | - Provide peer-support features while respect anonymity and privacy.  - Incorporate community support functions based on HCP feedback and local culture. |
